# The gut metagenomics and metabolomics signature in patients with inflammatory bowel disease

**DOI:** 10.1186/s13099-022-00499-9

**Published:** 2022-06-21

**Authors:** Xinwei Xu, Dickson Kofi Wiredu Ocansey, Sanhua Hang, Bo Wang, Samuel Amoah, Chengxue Yi, Xu Zhang, Lianqin Liu, Fei Mao

**Affiliations:** 1 Key Laboratory of Medical Science and Laboratory Medicine of Jiangsu Province, School of Medicine, Jiangsu University, Zhenjiang, 212013 Jiangsu People’s Republic of China; 2 The People’s Hospital of Danyang, Affiliated Danyang Hospital of Nantong University, Zhenjiang, 212300 Jiangsu People’s Republic of China; 3 Huai’an Maternity and Children Hospital, Huaian, 223002 Jiangsu People’s Republic of China; 4 Directorate of University Health Services, University of Cape Coast, PMB, Cape Coast, Ghana; 5 School of Medical Technology, Zhenjiang College, Zhenjiang, 212028 Jiangsu People’s Republic of China

**Keywords:** Inflammatory bowel disease, Metabolomics, Metagenomics, Gut bacteria, Differential metabolites

## Abstract

Inflammatory bowel disease (IBD), a chronic gut immune dysregulation and dysbiosis condition is rapidly increasing in global incidence. Regardless, there is a lack of ideal diagnostic markers, while conventional treatment provides scarce desired results, thus, the exploration for better options. Changes in the gut microbial composition and metabolites either lead to or are caused by the immune dysregulation that characterizes IBD. This study examined the fecal metagenomics and metabolomic changes in IBD patients. A total of 30 fecal samples were collected from 15 IBD patients and 15 healthy controls for 16S rDNA gene sequencing and UHPLC/Q-TOF-MS detection of metabolomics. Results showed that there was a severe perturbation of gut bacteria community composition, diversity, metabolites, and associated functions and metabolic pathways in IBD. This included a significantly decreased abundance of Bacteroidetes and Firmicutes, increased disease-associated phyla such as Proteobacteria and Actinobacteria, and increased *Escherichia* *coli* and *Klebsiella* *pneumoniae* in IBD. A total of 3146 metabolites were detected out of which 135 were differentially expressed between IBD and controls. Metabolites with high sensitivity and specificity in differentiating IBD from healthy individuals included 6,7,4′-trihydroxyisoflavone and thyroxine 4′-o-.beta.-d-glucuronide (AUC = 0.92), normorphine and salvinorin a (AUC = 0.90), and trichostachine (AUC = 0.91). Moreover, the IBD group had significantly affected pathways including primary bile acid biosynthesis, vitamin digestion and absorption, and carbohydrate metabolism. This study reveals that the combined evaluation of metabolites and fecal microbiome can be useful to discriminate between healthy subjects and IBD patients and consequently serve as therapeutic and diagnostic targets.

**Supplementary Information:**

The online version contains supplementary material available at 10.1186/s13099-022-00499-9.

## Introduction

IBD, consisting of ulcerative colitis (UC) and Crohn’s disease (CD), is a group of immunologically associated chronic disorders that primarily affect the gastrointestinal tract, with a high tendency to recrudesce in the lifetime. Up to date, the etiology and pathogenesis of IBD largely remain unclear, while the current documented hypothesis holds that the disease results from multifactorial interactions between genetic, microbial, environmental, and immunological elements [1]. Considering the constantly increasing prevalence in the developed countries, and the rapidly surging incidence in the developing countries, the disability-adjusted life year and burden of disease are on the increase with much global concern [2, 3]. This calls for intense study of IBD, on the quest to identifying not only therapeutic targets but also diagnostic and prognostic markers to improve disease management.

Among the gut factors that have shown promising therapeutic and diagnostic properties are the gut microbiome and metabolites. The human gut contains 1000–5000 different species of microbes, with approximately 99% coming from Firmicutes, Proteobacteria, Bacteroidetes, and Actinobacteria. In the IBD environment, chronic immune dysregulation is intertwined with the aberrant composition and diversity of these microbes (dysbiosis) and their metabolomics [4, 5]. The physiological role of the gut metagenomics and metabolomics, as well as their link with IBD pathogenesis, have been widely explored [6–9], where they are severely altered in the gut of IBD patients. Although UC and CD share many epidemiologic, immunologic, therapeutic, and clinical features, studies show that they have distinct profiles at the microbiome level [10]. The human gut microbiome represents a complex ecosystem contributing essential functions to its host. Although recent large-scale metagenomics studies have provided insights into its structure and functional potential, the functional repertoire which is contributed to human physiology and pathology remains largely unexplored [11], including in IBD. Moreover, the gut metabolite profile, which is jointly derived from microbially-derived compounds, diet, and modified human metabolites, shapes the microbiota-host interactions [12], thus, a crucial part of IBD pathogenesis.

Emerging studies around the cross point between IBD and gut metagenomics/metabolomics are promising and anticipated to soon impact daily medical practice significantly. This study examined the differential gut metagenomics and metabolomics profile between IBD patients and healthy controls from stool samples. The correlation between flora and metabolites of differential significance was also analyzed.

## Results

### Variations in gut bacteria community between IBD and healthy individuals

**Fig. 1:**
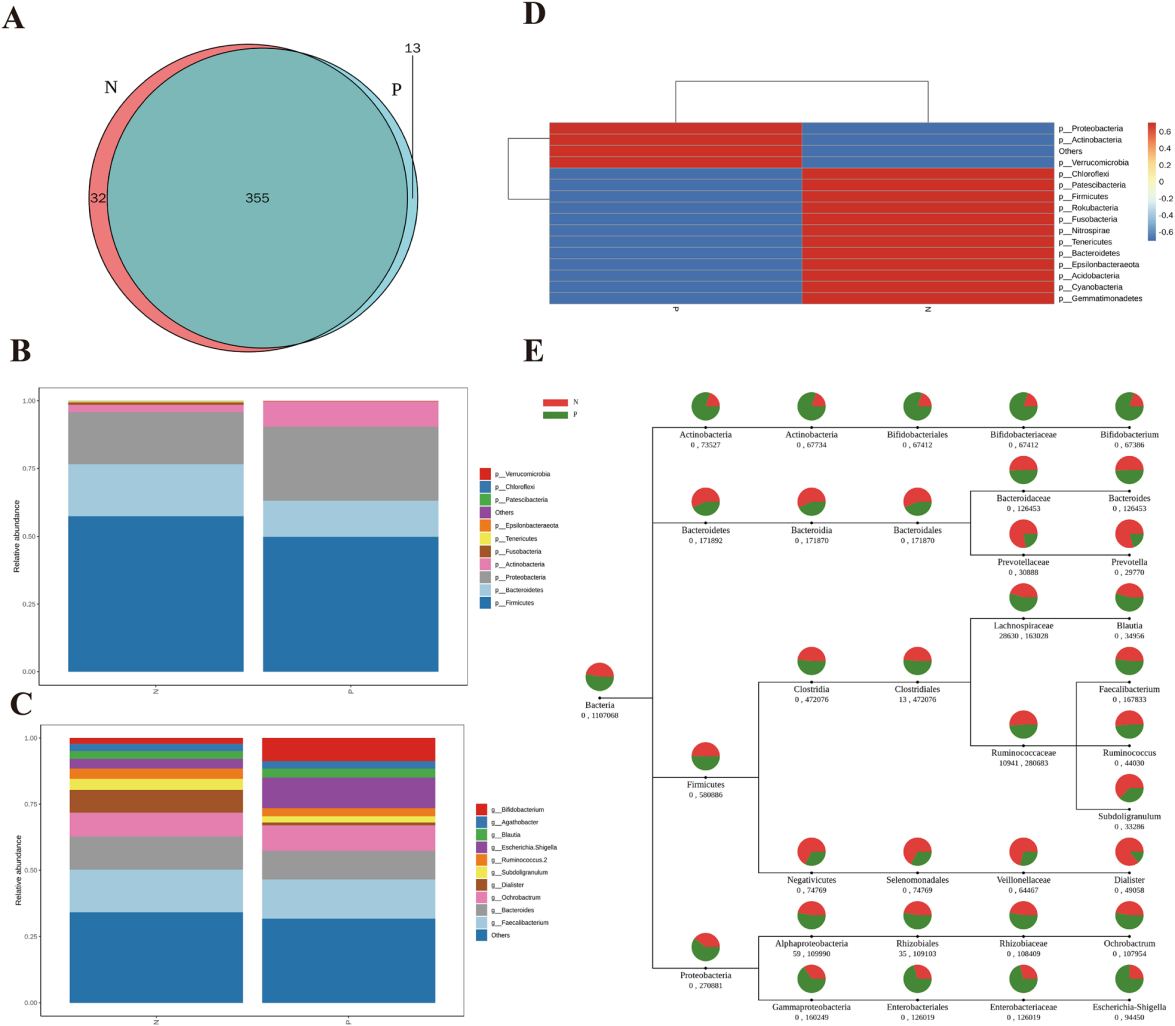
Gut bacteria community variations between IBD and healthy individuals. **A** Venn diagram; **B** Variation in the top 10 abundant phyla between groups; **C** Variation in the top 10 abundant species between the groups; **D** Community cluster heatmap at the phylum level; **E** Group specific species classification tree. N—Healthy control group; P—IBD group

As the most suitable index for bacterial phylogeny and taxonomic identification, 16S rDNA was used to assess differences in gut bacteria community between the two groups. Results showed significant variations in bacteria composition from the phylum to species levels. The exploration of OTUs via UCLUST in QIIME software revealed that while both groups shared a large proportion of the OTUs (355 common OTU’s), IBD samples had 13 unique OTUs and the control had 32 unique OTUs (Fig. 1A). At the phylum level, the bacteria community structure of IBD patients had reduced levels of Firmicutes, Bacteroides, Fusobacteria, and Tenericutes but increased abundance of Proteobacteria and Actinobacteria compared with healthy controls. The top 10 abundant phyla between the healthy and IBD groups are presented in Fig. 1B and Table 1. We further explored the specific bacteria alterations between the two groups by examining the top 10 species of significant abundance, where increased abundance in IBD included *Escherichia* *coli*, *Klebsiella* *pneumoniae*, *Bifidobacterium* *longum* subsp. Longum, *Bacteroides* *ovatus* V975, and uncultured bacterium, while uncultured Bacteroides sp. and s_gut metagenome/human gut metagenome were reduced in abundance (Table 1, Fig. 1C). Community Heatmap map was used to intuitively express the size of the clustered data value at each classification level. The phylum-level clustering in IBD confirmed a significantly increased abundance of Proteobacteria, Actinobacteria, and Verrucomicrobia and a decreased abundance of 12 other phyla as sown in Fig. 1D. Moreover, group specific species classification tree revealed the changes at all levels (Fig. 1E). For instance, at the genus level, IBD samples had reduced Bacteroides, Dialister, Subdoligradulum, and Ruminococcus 2, but increased abundance of Escherichia-Shigella and Bifidobacterium.

**Table 1:** Species annotation of the top 10 gut flora with the largest abundance in each group at the phyla and species classification levels

	Phylum name	Relative abundance in healthy controls	Relative abundance in IBD patients		Species name	Relative abundance in healthy controls	Relative abundance in IBD patients
Phylum	*Firmicutes*	0.573439	0.498548	Species	*Uncultured bacterium*	0.534105	0.446245
	*Proteobacteria*	0.192233	0.273626		*Uncultured organism*	0.095377	0.102373
	*Bacteroidetes*	0.19225	0.132276		*Escherichia coli*	0.036243	0.116567
	*Actinobacteria*	0.027346	0.092594		*Unidentified*	0.017717	0.071877
	*Fusobacteria*	0.008666	0.00015		*Gut metagenome*	0.078422	0.011995
	*Tenericutes*	0.004209	3.39E-05		*Klebsiella pneumoniae*	0.016731	0.026042
	*Verrucomicrobia*	0.000156	0.001754		*Bacteroides ovatus V975*	0.009631	0.023501
	*Epsilonbacteraeota*	0.000686	0.000407		*Bifidobacterium longum subsp. longum*	0.005054	0.017934
	*Patescibacteria* *Chloroflexi*	0.000274 0.000164	0.000214 8.63E-05		*Human gut metagenome*	0.021813	0.006999
					*Uncultured Bacteroides sp.*	0.001932	0.015503
	Others	0.000576	0.000311		Others	0.182975	0.160965

### Alpha- and beta-diversity changes in gut bacteria community in IBD patients

**Fig. 2:**
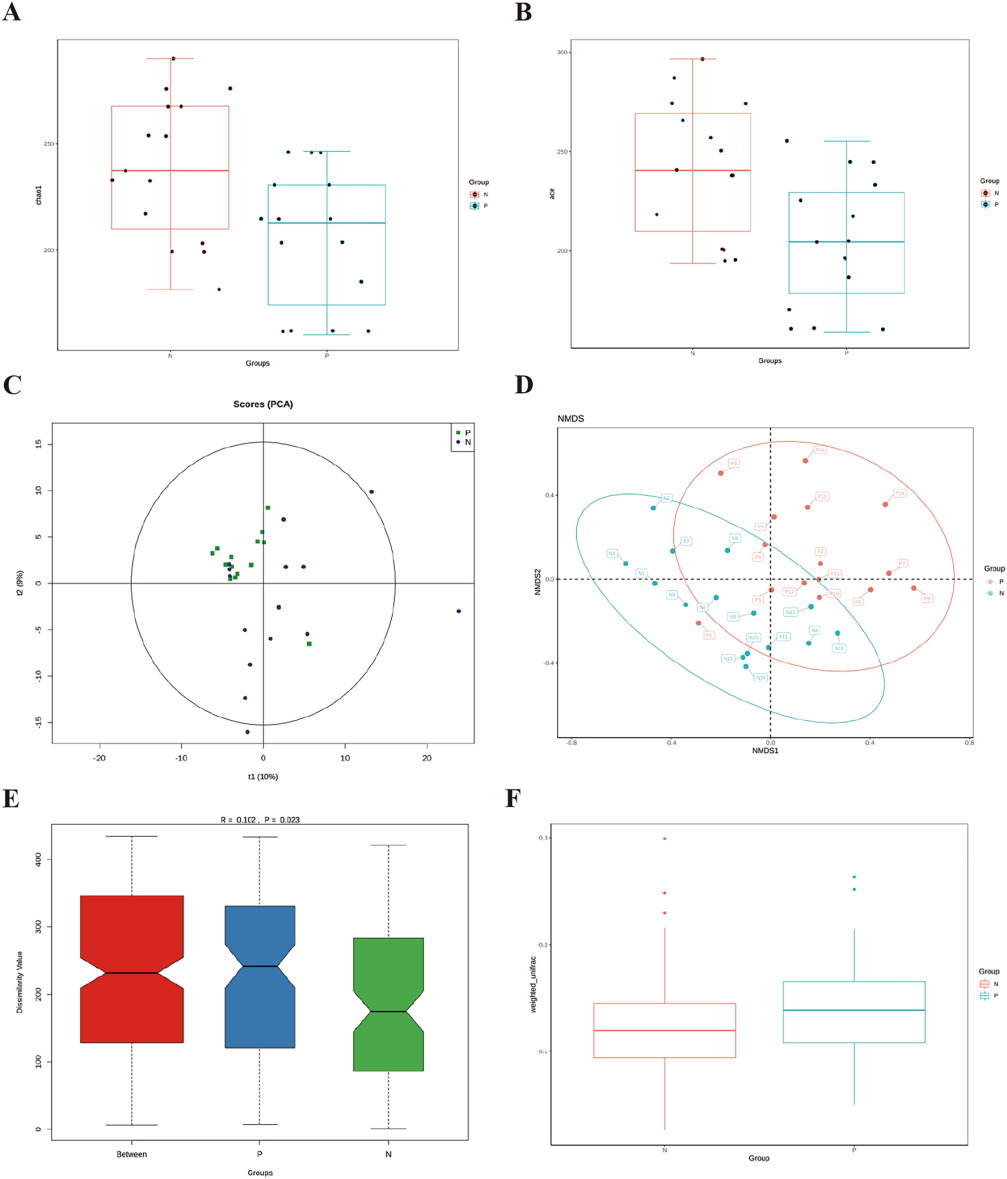
α and β diversity variation in the groups. **A** Chao 1 box chart of α diversity differences between the groups; **B** Abundance-based coverage estimator box chart of α diversity differences between the groups; **C** PCA of the community composition of the groups; **D** NMDS analysis reflecting the nonlinear structure of the bacteria community composition of the groups; **E** Anosim group differences in β diversity; **F** Weighted UniFrac distance box chart of β diversity differences between the groups. N—Healthy control group; P—IBD group

To explore the differences in α-diversity index between the groups, four diversity indexes (Chao 1, ACE [abundance-based coverage estimator], goods coverage, and observed species) were used. These tools revealed significant differences in the bacteria diversity between IBD samples and normal controls by intuitively reflecting the median, dispersion, maximum, minimum, and abnormal values of species diversity in the groups. There was significantly reduced α-diversity in IBD samples compared to healthy controls; Chao 1(p = 0.009), ACE (p = 0.004), goods coverage (p = 0.021), observed specifications (p = 0.002) (Fig. 2A, B). To further confirm the difference between the two sample groups to the greatest extent, principal component analysis (PCA) and non-metric multidimensional scaling (NMDS) statistics were employed. PCA results showed more closely clustered IBD samples, indicating reduced α-diversity as compared to the more scattered healthy control samples, indicating a more diverse bacterial community composition (Fig. 2C). The NMDS statistical ranking method, as a nonlinear model, was used to overcome the shortcomings of the linear model (i.e., PCA) and better reflect the nonlinear structure of data. The multi-dimensional space generated by NMDS revealed the degree of difference between both the inter—and intra- groups (Fig. 2D). In the analysis of β-diversity index differences between the groups, the nonparametric test, Anosim, revealed a significant difference in β diversity between the two groups (Fig. 2E). The weighted UniFrac distance box chart further confirmed the increased β-diversity in the IBD group (p = 0.005) (Fig. 2F).

### Biomarker analysis and functional prediction between IBD and healthy control

**Fig. 3:**
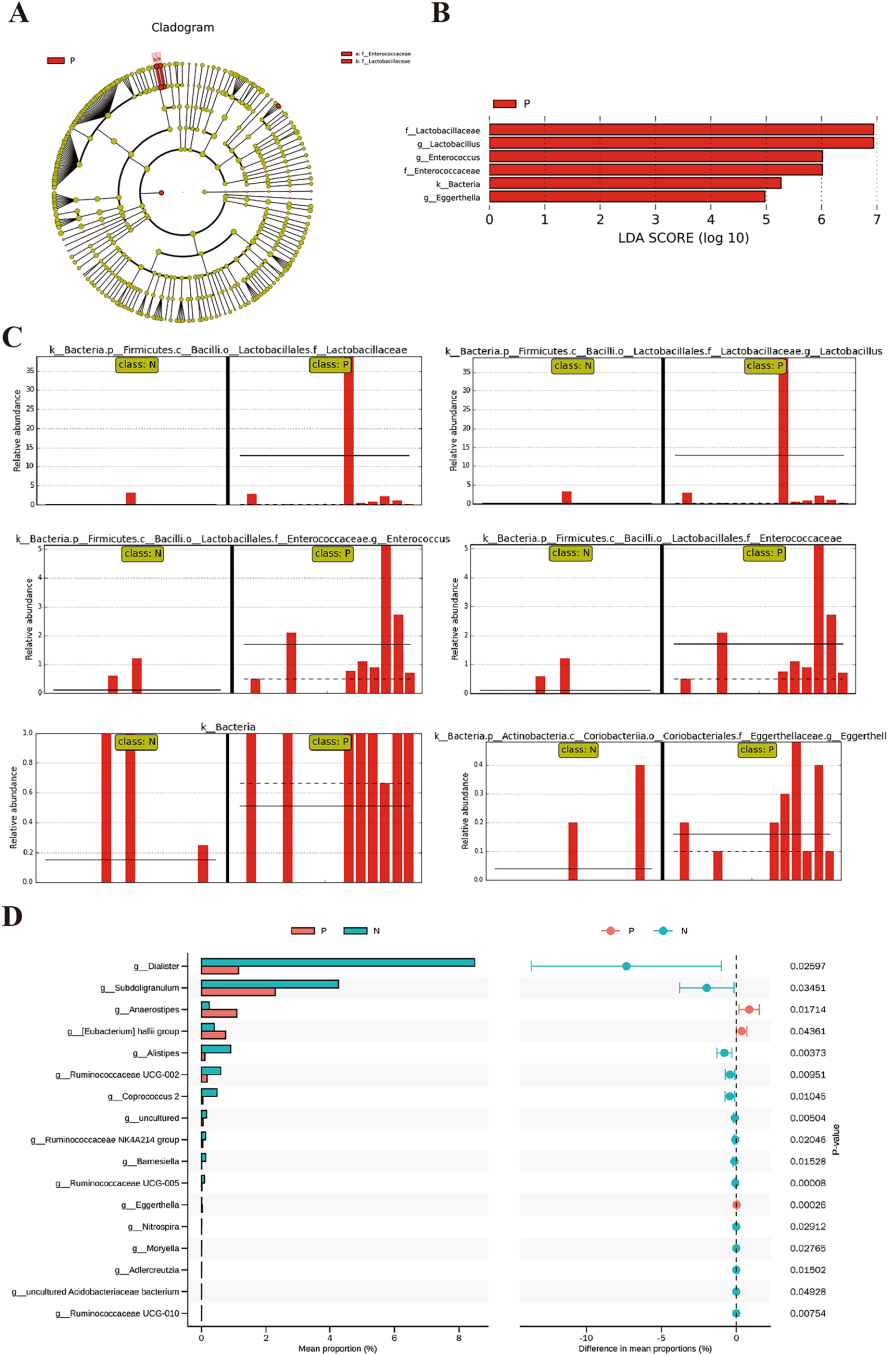
Microbial biomarker analysis between IBD and healthy controls. **A** Cladogram of LEFSe analysis results in the IBD group; **B** LDA value distribution differentiating IBD group; **C** Relative abundance of the potential biomarker in the IBD group; **D** STAMP differential analysis of bacterial populations between the groups at the genus level

The observed differences were further analyzed to discover possible high-dimensional biomarkers and genomic features that differentiate IBD stool samples from normal controls using the LEfSe software. The results, including a cladogram, linear discriminant analysis (LDA) value distribution, and abundance comparison diagram of biomarkers with statistical differences between the two groups, revealed probable biomarkers for IBD. There was increased abundance and genomic features of the families *Enterococcaceae* and *Lactobacillaceae* and the genera *Enterococcus, Lactobacillus*, and *Eggerthella,* representing the microbial groups that play an important role in the IBD group, and serving as distinguishing biomarkers (Fig. 3A–C). Moreover, STAMP differential analysis revealed several bacteria communities that significantly differentiate the IBD group from the healthy group at the genus level, including reduced relative abundance of *Dialister, Alistipes, Subdoligranulum, Ruminococcaceae UCG-002, UCG-005, UCG-010*, and *Coprococcus 2,* but increased abundance of *Anaerostipes, [Eubacterium] hallii group, and Eggerthella* (Fig. 3D).

**Fig. 4:**
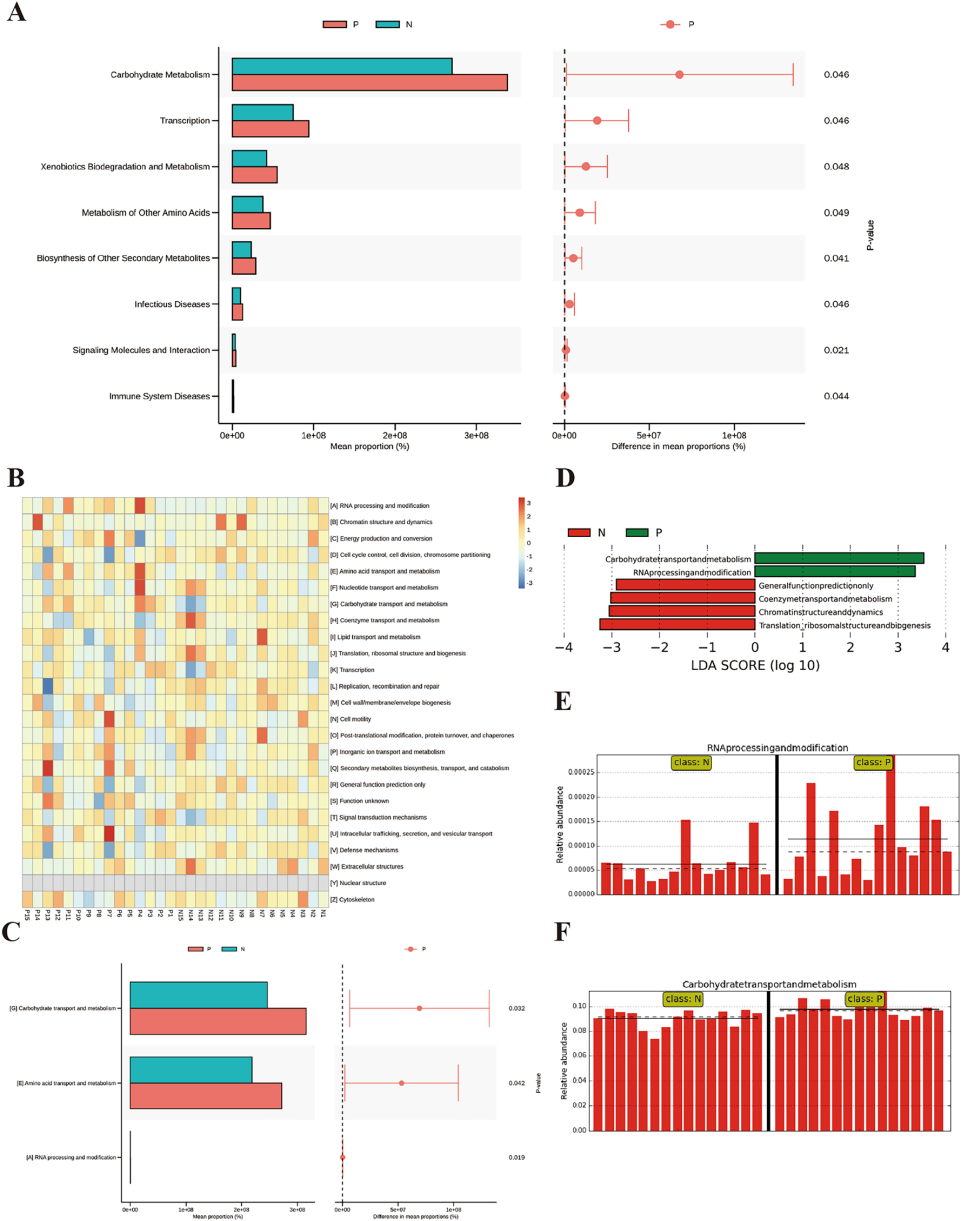
Functional prediction and biomarker analysis of the groups. **A** KEGG STAMP analysis of the significant gene composition variations between the groups; **B** COG heatmap analysis of the significant gene composition variations between the groups; **C** COG STAMP analysis of the significant gene composition variations between the groups; **D** LDA value distribution and comparison of the abundance of functional items with statistical differences between the groups based on COG function prediction; **E** The comparison of abundance of RNA processing and modification function; **F** The comparison of abundance of carbohydrate metabolism and transport function

For functional prediction in IBD and its differential value, the PICRUSt software was used to infer the functional gene composition of samples by comparing the species composition information obtained from 16S sequencing data, to analyze the functional differences between the different groups and their value as biomarkers. Moreover, the COG homologous protein cluster and function classification database of prokaryotes was used to complement KEGG and reveal the functional composition of the flora more comprehensively. The KEGG and COG function prediction analyses of the metabolic function changes in the IBD group via STAMP analysis showed significantly increased factors such as carbohydrate metabolism and transport, transcription, xenobiotics biodegradation and metabolism, metabolism and transport of amino acids, and biosynthesis of other secondary metabolites (Fig. 4A, C), as associated with the heatmap analysis of the significant gene composition variations between the groups (Fig. 4B). There was also increased functional indication of immune system diseases and infectious diseases in the IBD group (Fig. 4A). LEfSe LDA analysis based on COG homologous protein cluster and function classification revealed significantly elevated carbohydrate transport and metabolism and RNA processing and modification in the IBD group as against reduced translation of ribosomal structures and biogenesis, and chromatin structure and dynamics (Fig. 4D). The abundance comparison of the increased functional items (as appeared in individual samples) in the two groups is further shown in Fig. 4E, F.

### Variations in gut metabolomics between IBD and healthy controls

#### Differential analysis of significant metabolites

**Fig. 5:**
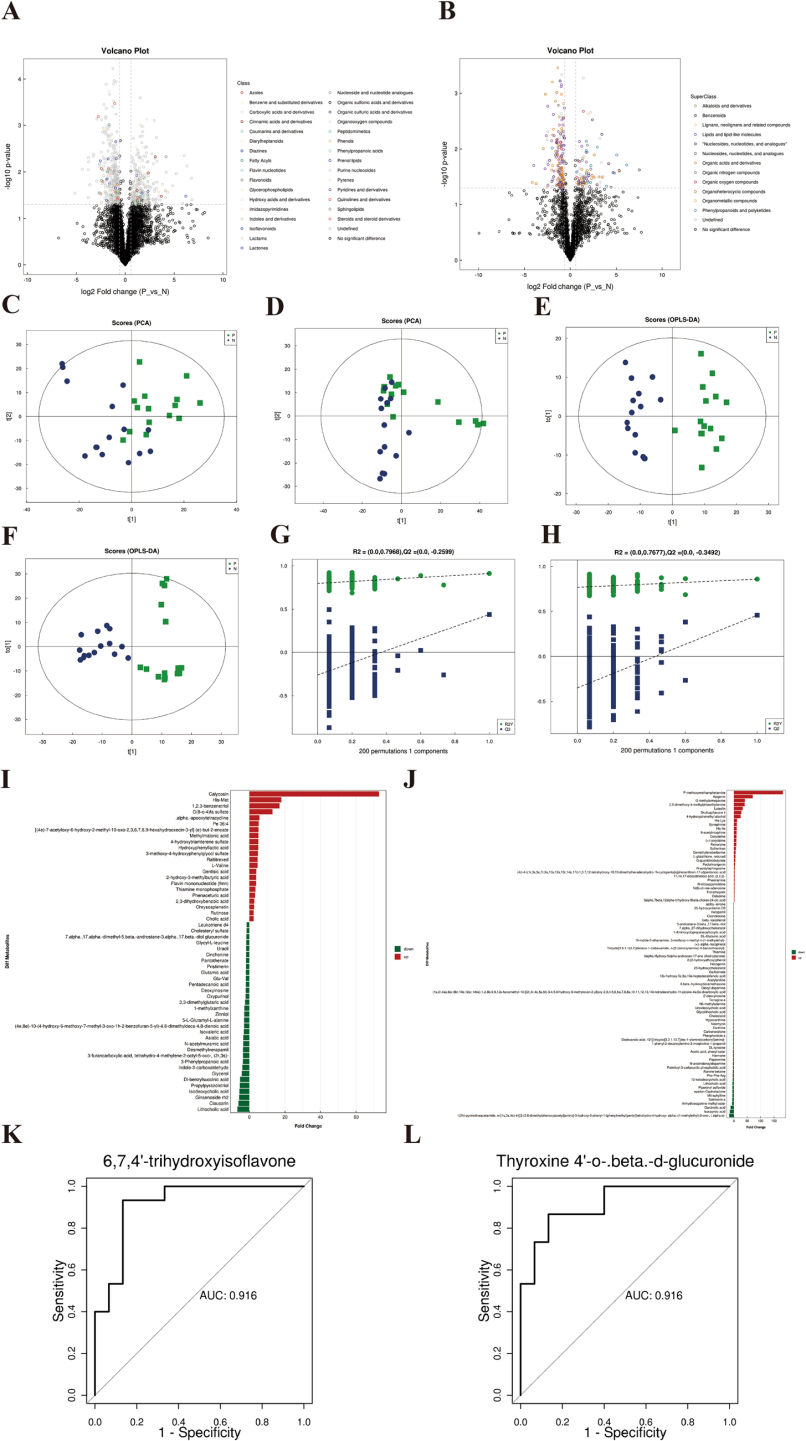
Differential analysis of significant metabolites between IBD and healthy controls. **A** Volcano plot of significantly different metabolites according to molecular class in negative ion mode; **B** Volcano plot of significantly different metabolites according to molecular class in positive ion mode; **C** PCA score diagram of negative ion mode; **D** PCA score diagram of positive ion mode; **E** Negative ion mode OPLS-DA score plot; **F** Positive ion mode OPLS-DA score plot; **G** Negative ion mode OPLS-DA displacement test; **H** Positive ion mode OPLS-DA displacement test; **I** Multiple analysis of significant differences in metabolite expression in negative ion mode; **J** Multiple analysis of significant differences in metabolite expression in positive ion mode; **K** AUC of 6,7,4'-trihydroxyisoflavone; **L** AUC of [(2r,3 s,4 s,5r,6r)-3,4,5-trihydroxy-6-[2-(3-hydroxy-5-oxooxolan-3-yl)propoxy]oxan-2-yl]methyl (e)-3-(3,4-dihydroxyphenyl)prop-2-enoate (0.91). N—Healthy control group; P—IBD group

A high-resolution nontargeted metabolomics analysis using ultra-high-performance liquid chromatography-quadrupole time-of-flight mass spectrometry (UHPLC-Q-TOF MS) was carried out to identify metabolites, followed by strict checks and manual confirmation of results. The positive ion mode identified 2223 metabolites while the negative ion mode identified 1063 metabolites, yielding a combined total of 3146 metabolites. Further analysis revealed a total of 135 differential metabolites between IBD and healthy controls (Table 2). Based on univariate analysis (fold change (FC) analysis), all metabolites detected in positive and negative ion modes were screened for the differential metabolites (FC > 1.5- rose red, FC < 0.67- blue, p-value < 0.05) in a volcano plot. The significant differential metabolites were distributed among 33 classes and 14 superclasses of compounds (Fig. 5A, B). PCA and orthogonal partial least squares discriminant analysis (OPLS-DA) of both the negative and positive ion mode (Fig. 5C–F) along with their displacement test (Fig. 5G, H) confirmed a distinct set of differential metabolites associated with the groups.

**Table 2:** An overview of the metabolomic analysis outcome

Key metabolomics analysis results	
*Total metabolites identified*	3146

Comparative analysis of differences between groups		
*Groups*	*Total differential metabolites*	*Significant difference metabolic pathways*
IBD vs healthy controls	135	Vitamin digestion and absorption, primary bile acid biosynthesis, protein digestion and absorption, ABC transporters, basal cell carcinoma, glutathione metabolism, ferroptosis

AUC aggregate measure of performance of differential metabolites	
*Metabolite*	*AUC value*
6,7,4′-trihydroxyisoflavone	0.92
Thyroxine 4′-o-.beta.-d-glucuronide	0.92
Trichostachine	0.91
[(2r,3 s,4 s,5r,6r)-3,4,5-trihydroxy-6-[2-(3-hydroxy-5-oxooxolan-3-yl)propoxy]oxan-2-yl]methyl (e)-3-(3,4-dihydroxyphenyl)prop-2-enoate	0.91
Normorphine	0.90
Salvinorin a	0.90
Esculetin	0.89
Mitraphylline	0.88
Indole-3-carboxaldehyde	0.88
Ginsenoside rh2	0.88
Arachidonoylserotonin	0.88
11-hydroxy-5z,8z,12e,14z,17z-eicosapentaenoic acid	0.88
10-deacetylbaccatin iii	0.88
9-cis-retinol	0.88
(5-benzoyloxy-3-chloro-4,6-dihydroxycyclohexen-1-yl)methyl benzoate	0.88
[(4e)-7-acetyloxy-6-hydroxy-2-methyl-10-oxo-2,3,6,7,8,9-hexahydrooxecin-3-yl] (e)-but-2-enoate	0.87
Dihydroberberine	0.87
N-nitrosopyrroolidine	0.87
Prothioconazole	0.87
Patchouli alcohol	0.87

In further examination of the differential metabolites, the negative ion mode molecules revealed the top five upregulated differential metabolites as Calycosin, His-Met, 1,2,3-benzenetriol, G(8-o-4)fa sulfate, and.Alpha.-apooxytetracycline, with the top five downregulated being Lithocholic acid, Clausarin, Ginsenoside rh2, Isodeoxycholic acid, and Propylpyrazoletriol (Fig. 5I). The top five upregulated versus downregulated differential metabolites in the positive ion mode were P-methoxymethamphetamine, Apigenin, O-methylarmepavine, 2,5-dimethoxy-4-methylphenethylamine, and Luteolin, versus 1(2 h)-pyrimidineacetamide, n-[(1 s,3 s,4 s)-4-[[2-(2,6-dimethylphenoxy)acetyl]amino]-3-hydroxy-5-phenyl-1-(phenylmethyl)pentyl]tetrahydro-4-hydroxy-.alpha.-(1-methylethyl)-2-oxo-, (.alpha.s)-, Isocaproic acid, Garcinolic acid, Anhydroecgonine methyl ester, and Salvinorin a, respectively (Fig. 5J). Furthermore, AUC (Area under the ROC Curve) analysis revealed several metabolites with high sensitivity and specificity in differentiating IBD from healthy individuals, including 6,7,4′-trihydroxyisoflavone (AUC = 0.92), thyroxine 4'-o-.beta.-d-glucuronide (AUC = 0.92), trichostachine (AUC = 0.91), normorphine (AUC = 0.90), and salvinorin a (AUC = 0.90). The top 20 metabolites in AUC measurement are presented in Table 2, while Fig. 5K, L shows representative diagrams of the AUC analysis.

#### Changes in metabolic pathways and function in IBD

**Fig. 6:**
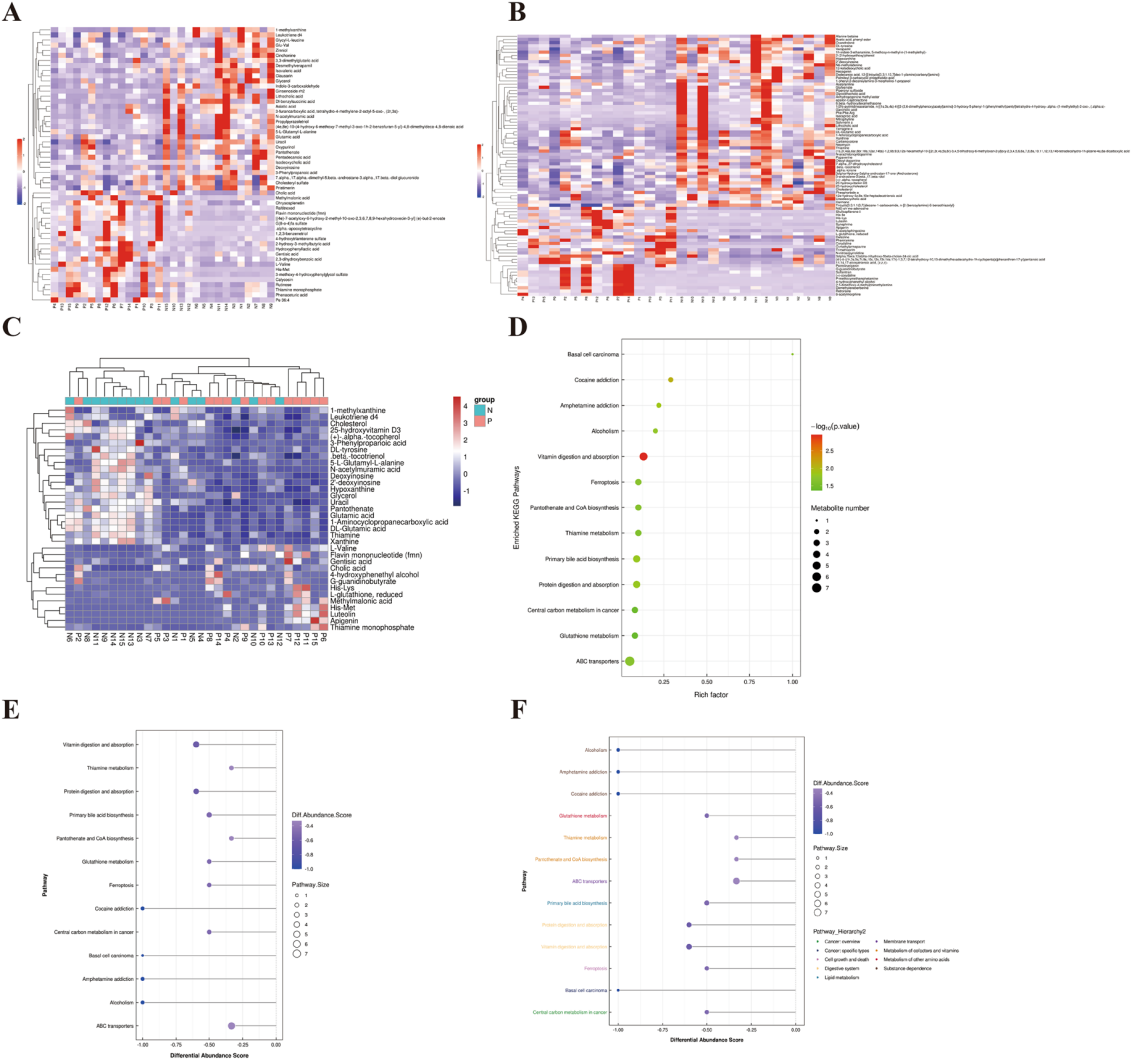
Changes in metabolic pathways and function. **A** Negative ion pattern of significantly different metabolite hierarchical clustering heat map of individual samples within the groups; **B** Positive ion pattern of significantly different metabolite hierarchical clustering heat map of individual samples within the groups; **C** KEGG pathway differential metabolite clustering heat map of ABC transport; **D** KEGG metabolic pathway enrichment map (Bubble chart); **E** Differential abundance score maps for all differential metabolic pathways; **F** Differential abundance score map of all differential metabolic pathways (classified according to pathway hierarchy). N—Healthy control group; P—IBD group

KEGG pathway enrichment analysis was carried out through the Fisher’s Exact Test to determine the significantly affected metabolic and signal transduction pathways in IBD. The results revealed altered metabolites (Fig. 6A, B) and 13 significantly affected pathways including vitamin digestion and absorption, primary bile acid biosynthesis, protein digestion, and absorption, thiamine metabolism, glutathione metabolism, ABC transporters, central carbon metabolism in cancer, and ferroptosis. The heatmap of differential metabolites in the largest pathway identified (ABC transport) is shown in Fig. 6C. Analysis of overall changes of KEGG metabolic pathway using differential abundance score and pathway enrichment is shown in Fig. 6D, E. Pathway hierarchy analysis showed that the changes in the IBD patients affected cancer function, cell growth and death, digestive system, lipid metabolism, membrane transport, and metabolism of cofactors, vitamins, and other amino acids (Fig. 6F). The specific metabolites dysregulated in these pathways are presented in Table 3. These results indicate significantly altered metabolomics and associated pathways in IBD patients compared to healthy individuals.

**Table 3:** Dysregulated KEGG metabolic pathways and associated metabolites in IBD

Pathway hierarchy	Map ID	Map name	Metabolite name	Up number	Down number
Digestive system	hsa04977	Vitamin digestion and absorption	Flavin mononucleotide (fmn), Pantothenate, Thiamine, ( +)-.alpha.-tocopherol, Cholesterol	1	4
Digestive system	hsa04974	Protein digestion and absorption	L-Valine, Isovaleric acid, Glutamic acid, DL-tyrosine, DL-Glutamic acid	1	4
Lipid metabolism	hsa00120	Primary bile acid biosynthesis	Cholic acid, 25-hydroxycholesterol, 7.alpha., 27-dihydroxycholesterol|Cholesterol	1	3
Cell growth and death	hsa04216	Ferroptosis	Glutamic acid, ( +)-.alpha.-tocopherol, DL-Glutamic acid, L-glutathione, reduced	1	3
Membrane transport	hsa02010	ABC transporters	L-Valine, Glycerol, Glutamic acid, Deoxyinosine|2′-deoxyinosine, Thiamine, DL-Glutamic acid, His-Lys, L-glutathione, reduced	3	6
Metabolism of cofactors and vitamins	hsa00770	Pantothenate and CoA biosynthesis	L-Valine, Uracil, Pantothenate	1	2
Metabolism of cofactors and vitamins	hsa00730	Thiamine metabolism	Thiamine monophosphate, DL-tyrosine, Thiamine	1	2
Cancer: overview	hsa05230	Central carbon metabolism in cancer	L-Valine, Glutamic acid, DL-tyrosine, DL-Glutamic acid	1	3
Metabolism of other amino acids	hsa00480	Glutathione metabolism	Glutamic acid, 5-L-Glutamyl-L-alanine, DL-Glutamic acid, L-glutathione, reduced	1	3

### Correlation of differential flora and metabolites in IBD

**Fig. 7:**
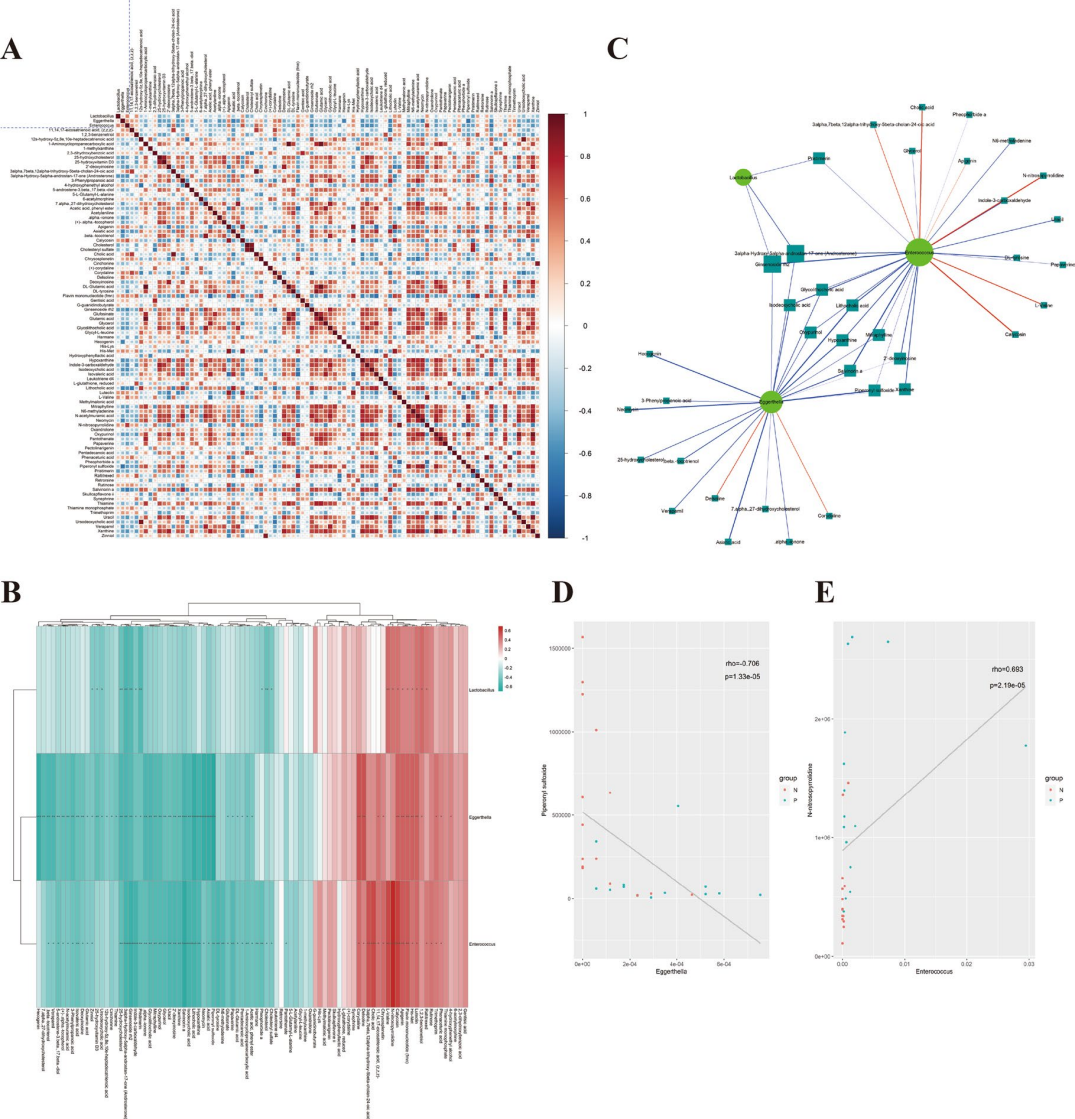
Association analysis of flora and metabolites with significant difference between the groups. **A** Spearman correlation coefficient matrix heat map of significant difference flora and metabolites; **B** Spearman correlation analysis hierarchical clustering heat map of significant difference flora and metabolites. The correlation coefficient R is expressed in color, where R > 0 indicates a positive correlation and is represented by red, R < 0 indicates a negative correlation and is expressed in blue. The darker the color, the stronger the correlation. P-value reflects the significant level of correlation and was defined by P < 0.05 as *, P < 0.01 as * *, P < 0.001 as * * *; **C** Correlation network diagram. The color of the line represents the positive and negative value of the correlation coefficient between the two (blue represents negative correlation and red represents positive correlation), and the thickness of the line is directly proportional to the absolute value of the correlation coefficient. The node size is positively correlated with its degree, that is, the greater the degree, the larger the node size. Spearman correlation analysis network of significant difference flora and metabolites; **D**, **E** Representative scatter diagram of correlation

To further assess the metabolomics changes in the IBD group, the relative abundance of three flora of significant difference at the genus level (Eggerthella, Enterococcus, Lactobacillus) and 89 significantly differential metabolites were sorted and analyzed. Spearman analysis was used to generate a correlation coefficient matrix heat map and hierarchical clustering heat map (Fig. 7A, B) to reflect the similarities and differences of expression patterns of the significant flora and metabolites. There were 1144 pairs of significantly related differential bacteria and metabolites, of which 285 pairs had a more significant correlation (P < 0.01). The matrix not only showed the correlation between significantly different flora and metabolites but also between significantly different metabolites-metabolite and flora-flora. Enterococcus had positive significant correlation with 17 metabolites including cholic acid, calycosin, and N-nitrosopyrrolidine (p < 0.001), and flavin mononucleotide, apigenin, L-valine, and 3alpha,7beta,12alpha-trihydroxy-5beta-cholan-24-oic acid (p < 0.01), but negatively significant correlation with 43 metabolites including ginsenoside rh2, androsterone, indole-3-carboxaldehyde, salvinorin a, isodeoxycholic acid, and lithocholic acid (p < 0.001), and glycerol, uracil, oxypurinol, 25-hydroxycholesterol, glycolithocholic aid, xanthine, and hypoxyxanthine (p < 0.01). Eggerthella positively correlated with 13 metabolites including corydaline, delsoline, calycosin, apigenin, flavin mononucleotide, his-met, and luteolin (p < 0.01), but negatively correlated with 43 metabolites including hecogenin, salvinorin a, lithocholic acid, hypoxanthine, neomycin, Asiatic acid, piperonyl sulfoxide (p < 0.001). Lactobacillus had positive significant correlation with 9 metabolites including L-valine (p < 0.001), N-nitrosopyrrolidine, calycosin, apigenin, flavin mononucleotide, his-met, luteolin, and 1,2,3-benzenetriol (p < 0.05), but negative significant correlation with 11 metabolites including 25-hydroxycholesterol, androsterone, ginsenoside rh2, pristimerin, and cholesterol (p < 0.01) (Fig. 7B).

Moreover, the Cytoscape 3.5.1 software was used to generate a different perspective of the relationship between the flora and metabolites. The network chart revealed a total of 8 pairs of flora-metabolites with significant positive correlation and 44 pairs with a significant negative correlation that connect the three flora (Fig. 7C). The distribution characteristics of the correlation were also generated with a scatter diagram, which revealed 52 pairs of correlated flora-metabolites with significant levels. For example, the scatter diagram of the correlation between Eggerthella and piperonyl sulfoxide, and Enterococcus and N-nitrosopyrrolidine are shown in Figs. 7D, E. These observations do not only reveal changes in IBD but also provide important data in the search for therapeutic targets and diagnostic markers in IBD. However, more specific and detailed studies are required.

## Discussion

The role of the intestinal microbiota in human health continues to gain more research attention since changes in the composition of the intestinal bacterial community or environment have been demonstrated in patients with diseases such as IBD, neurodegenerative diseases, cancers, allergy, autoimmune diseases, as well as some lifestyle-related and metabolic diseases [13–15]. A healthy gut environment is regulated by the exquisite balance of intestinal microbiota, metabolites, and the host's immune system. Host physiology can be altered at the cellular level by microbiome-induced cell signaling, proliferation, and neurotransmitter biosynthesis, leading to mucosal and systemic alterations and thereby affecting homeostasis, barrier function, innate and adaptive immune responses, and metabolism [16]. With such a broad range of effects on host physiology and its role in the induction, education, and function of the immune system, it is not surprising that the microbiota is implicated in gut-related diseases including IBD. In this study, fecal samples were obtained from IBD and healthy adults to ascertain the alterations in the gut metagenomics profile of IBD patients. It was confirmed that IBD patients suffered severe perturbation in the gut bacteria community compared to healthy individuals. The two most abundant phyla in humans (Bacteroidetes and Firmicutes) were decreased while disease-associated phyla such as Proteobacteria and Actinobacteria were increased. The IBD samples were associated with an increased abundance of species such as *Escherichia* *coli*, *Klebsiella* *pneumoniae*, *Bifidobacterium* *longum* subsp. Longum, *Bacteroides* *ovatus* V975, and uncultured bacterium. Moreover, there was significantly altered alpha- and beta-diversity in the gut bacteria community in IBD patients.

These findings are confirmed by several studies including collective studies that found a decrease in gut microbial diversity in IBD patients with a decrease in Firmicutes [17, 18]. Matsuoka and Kanai [19] stated that the most consistent observation in IBD dysbiosis is reduced bacterial diversity; an increase of Proteobacteria and a decrease of Firmicutes [19]. A study of the composition of the microbiota and the metabolites in the stool of 183 subjects (82 UC, 50 CD, and 51 healthy controls) also revealed significantly increased Proteobacteria, Verrucomicrobia, and Fusobacteria but decreased Bacteroidetes and Cyanobacteria [20]. While bacteria species such as *Escherichia* *coli* are notable culprits in the causation and progression of gastrointestinal tract diseases, recent studies have recognized a new player, *Klebsiella* *pneumoniae*, in gastrointestinal tract disturbances [21] and as a dysbiosis-associated species in IBD [22]. In other studies, metagenomics and culturomics have identified strains of *Escherichia* *coli* and *Ruminococcus* *gnavus* to be linked to IBD and gut inflammation [23, 24]. It is also reported that the combination of Ruminococcaceae *F. prausnitzii* phylogroups and *Escherichia* *coli* offers the potential to discriminate between IBD and CRC patients and could assist in IBD subtypes classification [25]. The integration of these species may yield a potential biomarker for IBD diagnosis, thus, the need for further consideration. On the other hand, the increased abundance of *Bifidobacterium* *longum* subsp. Longum in the IBD samples may be attributed to host-responsive mechanisms against gut inflammation since this species has been demonstrated to possess strong antioxidant capacity [26], attenuate intestinal injury [27], and generally protect against IBD [28]. Alterations in the bacteria population is also linked with patients’ response to treatment as demonstrated by studies such as Dovrolis et al. [29], which reported that Infliximab treatment has a notable impact on both the gut microbial composition and the inflamed tissue transcriptome in IBD patients [29].

The discovery of a reliable biomarker for IBD would be a breakthrough for the disease diagnostic and possible treatment. The study, therefore, examined the observed bacteria differences for further discovery of possible high-dimensional biomarkers and genomic features that differentiate IBD stool samples from normal controls. The genera Enterococcus, Lactobacillus, and Eggerthella, representing the microbial groups that play an important role in the IBD group, served as distinguishing biomarkers. Other potential biomarkers for IBD include elevated abundance of Anaerostipes and [Eubacterium] hallii group, and reduced population of Ruminococcaceae UCG-002, UCG-005, UCG-010, Coprococcus 2, Dialister, Alistipes, and Subdoligranulum. However, these observations require further detailed exploration. Studies that agree with this finding include a recent study on functional dysbiosis in the gut microbiome during IBD activity, which demonstrated a characteristic increase in facultative anaerobes at the expense of obligate anaerobes. For example, the relative abundance of Ruminococcaceae UCG 005 and *Eubacterium* *rectale* decreased with increasing IBD-associated host-microbial interaction factors [22]. Most of the decreased bacteria population possess anti-inflammation properties, thus an indication of compromised inflammation resolution. For instance, the Ruminococcaceae, *Faecalibacterium prausnitzii*, which is the most abundant bacterium in the human intestinal microbiota of healthy adults (representing more than 5% of the total bacterial population) is depleted in CD and UC and has been shown to have in vitro and in vivo anti-inflammatory properties [30]. Alterations in Lachnospiraceae and Ruminococcaceae families in both CD and UC patients, typical producers of short-chain fatty acids, characterize frequently relapsing disease and poor responses to treatment, as well as the risk of later disease recurrence of patients in remission [31]. Another study reported significantly reduced abundance of *Faecalibacterium prausnitzii* and *Eubacterium rectale*, but enriched *Escherichia coli* in UC patients, where *Escherichia coli* abundance correlated positively with increased abundance of several virulence genes [32].

It has been reported that metagenomics approaches alone are insufficient to infer the functional metabolic activity of the microbiome [33]. Thus, functional, pathway-based analyses are required to elucidate the changes in the composition of the gut microbiomes of IBD patients and the metabolic changes that could serve as a target for therapeutic interventions. Gut microbes can alter pools of available metabolites thereby modifying host-generated signaling molecules. This study applied the PICRUSt software to infer the functional gene composition of samples by comparing the species composition information obtained from 16S sequencing data, to analyze the functional differences between the different groups and their value as biomarkers. The COG and KEGG functional prediction analyses of the metabolic function changes in the IBD samples via STAMP showed significantly increased functions such as carbohydrate metabolism and transport, metabolism and transport of amino acids, transcription, xenobiotics biodegradation and metabolism, and biosynthesis of other secondary metabolites. There was also increased functional indication of immune system diseases and infectious diseases in the IBD group. Moreover, translation of ribosomal structures and biogenesis, and chromatin structure and dynamics were decreased in IBD. Liang [34] reported that the microbial and metabolic signatures of IBD patients are significantly different from those of healthy controls, and identified a total of 17 discriminative pathways between the two groups, mainly involved in amino acid, nucleotide biosynthesis, and carbohydrate degradation [34]. The gene expression signature of the colonic mucosa of UC patients showed dysregulation in mediators associated with carbohydrate metabolism, solute transport, autophagy, ubiquitination, ER stress, oxidative stress, and T cell regulation [35].

The last few years have seen an increase in the studies of experimental and human IBD focusing on the search for small metabolites, such as amino acids, bases, and tricarboxylic acid cycle intermediates. Experimental methods for the screening of metabolites including fecal extracts have shown that IBD patients and healthy individuals, as well as the IBD subtypes, express distinct metabolic profiles. Metabolomics data of fecal extracts have revealed disruptions in not only metabolites but bacterial populations, findings that are indicative of a close association between the two factors and their possible involvement in the development of IBDs [36, 37]. Researchers agree that a useful approach to gaining insight into the metabolic activity of a system is metabolomics measurements since metabolite profiles are a readout of what is happening at the biochemical level. Several studies have analyzed the fecal metabolome in IBD patients and cohorts and confirmed severe alterations compared with healthy individuals [12, 20, 38]. For instance, IBD patients have reduced fecal levels of the short-chain fatty acid butyrate, fecal medium-chain fatty acids (e.g., pentanoate and hexanoate), and fecal vitamin B levels, while fecal levels of lipids, amino acids, and amines have been reported to increase in IBD patients [20, 39]. In this study, UHPLC-Q-TOF MS identified a total of 3146 metabolites, out of which 135 were differentially expressed between IBD and healthy controls. The results of KEGG pathway enrichment analysis of the differential metabolites revealed 13 significantly affected pathways including generally decreased vitamin digestion and absorption, primary bile acid biosynthesis, protein digestion and absorption, ABC transporters, central carbon metabolism in cancer, glutathione metabolism, and ferroptosis.

Several untargeted studies have demonstrated huge disturbances of the gut metabolome in IBD, which is in keeping with the known dysbiosis in gut communities. Metabolite groups of interest include SCFAs, bile acid metabolites, vitamins, and tryptophan metabolites, where the essential roles for these metabolites in normal immune development, homeostasis, and IBD have been demonstrated [40]. It is documented that IBD patients suffer a significant risk of vitamin B12 and folate insufficiencies [41], vitamin D deficiency [42], among other micronutrient absorption and related outcomes [43]. Primary bile acids possess amphipathic properties, rendering them highly instrumental for not only lipid digestion and absorption, but immune responses and several metabolic functions in the small intestine [44, 45]. In the IBD group, differentially abundant species and functions from the metagenomics profiles reflected adaptation to oxidative stress in the IBD gut and are consistent with previous findings [12]. The dysregulation in glutathione metabolism, the most important intracellular antioxidant, may contribute to reactive oxygen species build-up, causing tissue injury in IBD as earlier reported [46]. Moreover, ferroptosis, a newly characterized form of regulated cell death, is driven by the lethal accumulation of lipid peroxides catalyzed by cellular free iron. It has been widely documented that the fundamental features of ferroptosis, including iron deposition, glutathione exhaustion, glutathione peroxidase 4 inactivation, and lipid peroxidation, are manifested in the injured gastrointestinal tract in IBD patients [47]. The dysregulation of these key functional pathways including protein digestion and absorption, and ABC transporters as a characteristic feature of IBD, contributes to the recurrent immune perturbation and subsequent tissue injury. These results underline the potential role of an inter-omics approach in understanding the metabolic pathways involved in IBD.

Concerning the mechanisms associated with the observed changes, it is reported that host genetics, immune dysregulation, and gut microbiota are broadly implicated. Genes such as NOD2, IRGM, ATG16L1, LRRK2, PTPN2, IL23R, Il10, Il10RA, Il10RB, CDH1, and HNF4α influence intestinal microbiome and metabolites in IBD [48]. For example, the CD polymorphism, ATG16L1 T300A, alters the gut microbiota and enhances the local Th1/Th17 response, contributing to dysbiosis and immune infiltration prior to disease symptoms [49]. Mucin-type O-glycans alter the diversity of gastrointestinal microorganisms, which in turn increases the level of O-glycosylation of host intestinal proteins via the utilization of glycans. The mechanism that influences the selection of host’s bacteria might involve mucin-type O-glycans as demonstrated in mice with Core-1 glycan deficiency in the small intestine, exhibiting higher levels of Bacteroidetes and lower levels of Firmicutes than wild-type mice [50], and in mice lacking β1, 4-N-acetylgalactosamine transferase 2 (B4galnt2) [51]. Microbial metabolites, including short chain fatty acids (SCFAs), tryptophan (Trp), bile acid, and vitamins are actively absorbed or diffused across the intestinal lining to affect the host response in the intestine as well as at systemic sites via the engagement of cognate receptors, influencing epithelial barrier function and intestinal homeostasis. In addition, food constituents such as micronutrients are important regulators of mucosal immunity, with direct or indirect effects on the gut microbiota, thus [52]. These findings indicate the complex molecular interaction between host’s immunity, genetics, and environmental factors in influencing gut microbiota and metabolites in IBD.

## Conclusion

The study reveals that IBD patients have severe perturbation of gut bacteria community composition, diversity, metabolites, and associated functions and metabolic pathways compared to healthy individuals. This indicates that the combined evaluation of metabolites and fecal microbiome can be useful to discriminate between healthy subjects and patients with IBD and consequently serve as therapeutic targets. However, the sample size of the study was small and was mainly adult UC patients, thus, further larger studies involving both UC ad CD patients of all age groups are required to examine the molecular signature of the differentially expressed metabolite and flora in the IBD group, since this could lead to the discovery of a novel diagnostic and therapeutic target of IBD. Again, this proof-of-concept approach prompts further investigation and detailed data mining of the correlation between the significantly differential metagenomics and metabolomics.

## Methods

### Human subjects and sample collection

The study was approved by the Ethical Committee of Jiangsu University (2,012,258). All human subjects agreed to participate in the study and were made to sign consent forms.

To assess the alterations in the gut metagenomics and metabolomics profile of IBD patients, fecal samples were obtained from 30 adults, made up of 15 confirmed IBD patients and 15 healthy individuals in the Huai ’an Hospital of Traditional Chinese Medicine, Jiangsu Province, China. All the 15 IBD patients were of the subtype UC, with 1 patient having an extra characteristic of colonic polyp () and 3 patients with chronic colitis (). The clinical diagnosis of the IBD patients was based on the consensus opinions on diagnosis and treatment of IBD (Beijing, 2018). The samples collected in this study were all in the remission stage of the disease. We matched healthy subjects with IBD patients by age, lifestyle, disease history, etc. All IBD patients had no disease history except two patients with hypertension. The mean ages of the healthy controls and IBD patients were 52.6 ± 2.7 years and 53.4 ± 3.6 years, respectively. The age, cholesterol level, blood glucose level, diabetes, hypertension, coronary heart disease, hepatitis, tuberculosis, traumatic surgery, poisoning, blood product transfusion history and other information of subjects in the two groups were basically the same.

### Metagenomics analysis

#### Bacteria community and predicted functions analysis

The experimental process for the fecal analysis of the bacteria community and predicted functions (metagenetic) involved six key stages (Additional file 1: Fig. S1A). From DNA extraction to computer sequencing, the sample quality was strictly controlled in each link to ensure the authenticity of sequencing data. For the combined analysis of 16S metabolomics, the process involved 16S rDNA amplicon sequencing of significantly different flora and significantly different metabolites, followed by different correlation analyses (Additional file 1: Fig. S1B).

#### OTU clustering, distribution, and species annotation

In order to study the species composition diversity of samples, the clean reads of all samples were clustered. Using UCLUST in QIIME (version 1.8.0) software, the clean reads were first de-chimerized, followed by clustering of the non-repetitive sequences into OTUs (operational taxonomic units) with 97% consistency, and then annotation of the representative sequences of OTUs using the Greenenes or Silva database. To avoid the interference caused by the difference of total sequencing quantity, some samples were leveled according to the minimum value of sequence number in each group, and the sequence number of all samples was randomly selected to a unified data volume for subsequent analysis. This process is illustrated in Additional file 1: Fig. S1C.

### Test of the adequacy of sample size and reliability of microbial information from the data

In addition to the quality control checks on all samples, several tools were used to assess the ability of the samples to present a true reflection and reliable information on the microbial community variability between the two groups. A rarefaction curve was used to randomly select a certain amount of sequencing data from samples, count the number of species they represent, and build a curve based on the amount of sequencing data extracted and the number of corresponding species. The resultant curve, directly and indirectly, reflected the reasonability/rationality of the amount of sequencing data and the richness of species in the samples respectively (Additional file 2: Fig. S2A). Moreover, a Shannon curve was constructed according to the microbial diversity index of the sequencing quantity of each sample at different sequencing depths. The flat Shannon curve produced indicated that the amount of sequencing data is large enough to reflect the vast majority of microbial information in the sample (Additional file 2: Fig. S2B). Rank abundance curve was used to assess two aspects of the bacterial diversity, namely species abundance and species evenness. Results indicated an adequate abundance of species as reflected by the larger range of the width of the curve on the horizontal axis, and the evenness of species in the sample as indicated by the smoothness of the shape of the curve (Additional file 2: Fig. S2C). As an effective tool to investigate species composition and predict the species abundance in samples, a species accumulation curve was employed to further judge the adequacy of sample size and estimate species richness. This analysis did not only confirm the sufficiency of the sample size, but also the species richness on the premise of sufficient sample size (Additional file 2: Fig. S2D).

### Metabolomics analysis

#### LC–MS/MS analysis of metabolomics

A high-resolution nontargeted metabolomics analysis of fecal samples was performed. Ultra-high performance liquid chromatography-quadrupole time-of-flight mass spectrometry (UHPLC/Q-TOF–MS) technique was used to detect metabolites in samples, which was matched with the retention time, molecular weight (molecular weight error < 25 ppm), secondary fragmentation spectrum, collision energy, and other information of metabolites in the local database, The structure of metabolites in biological samples was identified, and the identification results were strictly checked and confirmed manually.

#### Metabolomics data processing

The raw MS data (wiff.scan files) were converted to MzXML files using ProteoWizard MSConvert before importing into freely available XCMS software. For peak picking, the following parameters were used: centWave m/z = 25 ppm, peak width = c (10, 60), prefilter = c (10, 100). For peak grouping, bw = 5, mzwid = 0.025, minfrac = 0.5 were used. CAMERA (Collection of Algorithms of MEtabolite pRofile Annotation) was sued for annotation of isotopes and adducts. In the extracted ion features, only the variables having more than 50% of the nonzero measurement values in at least one group were kept. Compound identification of metabolites was performed by comparing accuracy m/z value (< 25 ppm), and MS/MS spectra with an in-house database established with available authentic standards.

### Combined analysis of 16S metabolomics (intestinal microbiological association analysis)

After 16S rDNA amplicon sequencing and analysis of fecal metagenomics and metabolomics, intestinal microbiological association analysis was also performed. This constitutes data in-depth mining, which helped to further depict the association or correlation hidden in the data set. The association analysis between 16S and metabolism used a statistical algorithm to find the association between the three significantly different flora and 89 significantly different metabolites.

The relative abundance (LEfSe LDA > 2 and P-value < 0.05) of three flora with a significant difference at genus level and the expression of 89 metabolites with significant difference (VIP > 1 and P-value < 0.05 of *t*-test) obtained by metabolomics analysis in all experimental samples were sorted in a table as the input file for subsequent analysis. Spearman statistical method was used to analyze the correlation coefficient between significantly different flora and metabolites screened in the experimental samples. Furthermore, matrix heat map, hierarchical clustering heat map, and correlation network were analyzed in combination with R language (R 3.4.2 Heatmap package) and Cytoscape software to explore the interaction relationship between flora and metabolites from multiple angles.

### Statistical analysis

To confirm differences in the abundances of individual taxonomy between the two groups, STAMP software was utilized. LEfSe was used for the quantitative analysis of biomarkers within different groups. To identify differences in microbial communities between the two groups, ANOSIM and ADONIS were performed based on the Bray–Curtis dissimilarity distance matrices. Other analyses performed on the metagenomics/metabolomics included PCA and multivariate statistical analysis using SIMCA Version 14.1, Pearson correlation analysis using CytoScape Version 3.5.1, and KEGG pathway analysis using R Version 3.5.1. P-value reflects the significant level of correlation and was defined by p < 0.05 as *, p < 0.01 as * *, p < 0.001 as * * *.

## Supplementary Information

**Additional file 1: Figure S1.** The 16S rDNA amplicon sequencing and data analysis flow chart A: 16S rDNA amplicon sequencing technology flow chart; B: Combined 16S rDNA amplicon sequencing of significantly different flora and significantly different metabolites; C: Data analysis process.

**Additional file 2: Figure S2.** Test of the adequacy of sample size and data reliability of microbial information; A: Rarefaction curve reflecting the rationality of the data and the richness of species in the sample; B: Shannon curve indicating that the amount of sequencing data is large enough to reflect the vast majority of microbial information in the samples; C: Rank abundance curve reflecting species abundance and uniform distribution of species; D: Species accumulation curve on the adequacy of sample size and estimation of species richness.

## Data Availability

The datasets used and/or analysed during the current study are available from the corresponding author on reasonable request.
