# Supplementary figures and images for: The gut metagenomics and metabolomics signature in patients with inflammatory bowel disease
Source: Gut Pathog. 2022 Jun 21;14:26. doi: 10.1186/s13099-022-00499-9 (PMC9215062; doi:10.1186/s13099-022-00499-9)

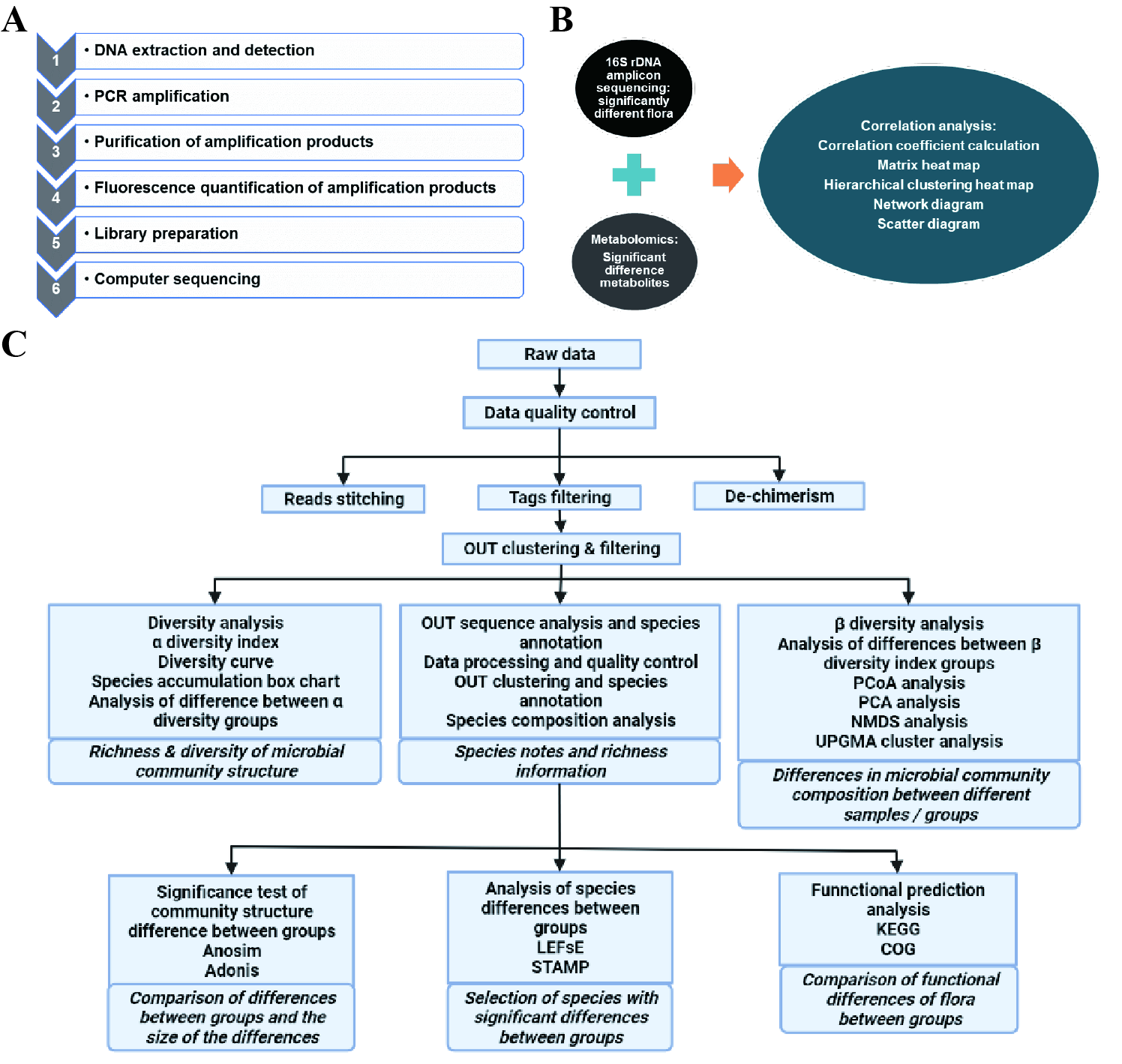

Supplement: Supplementary file 1 — Additional file 1: Figure S1. The 16S rDNA amplicon sequencing and data analysis flow chart A: 16S rDNA amplicon sequencing technology flow chart; B: Combined 16S rDNA amplicon sequencing of significantly different flora and significantly different metabolites; C: Data analysis process. [file 13099_2022_499_MOESM1_ESM.tif]

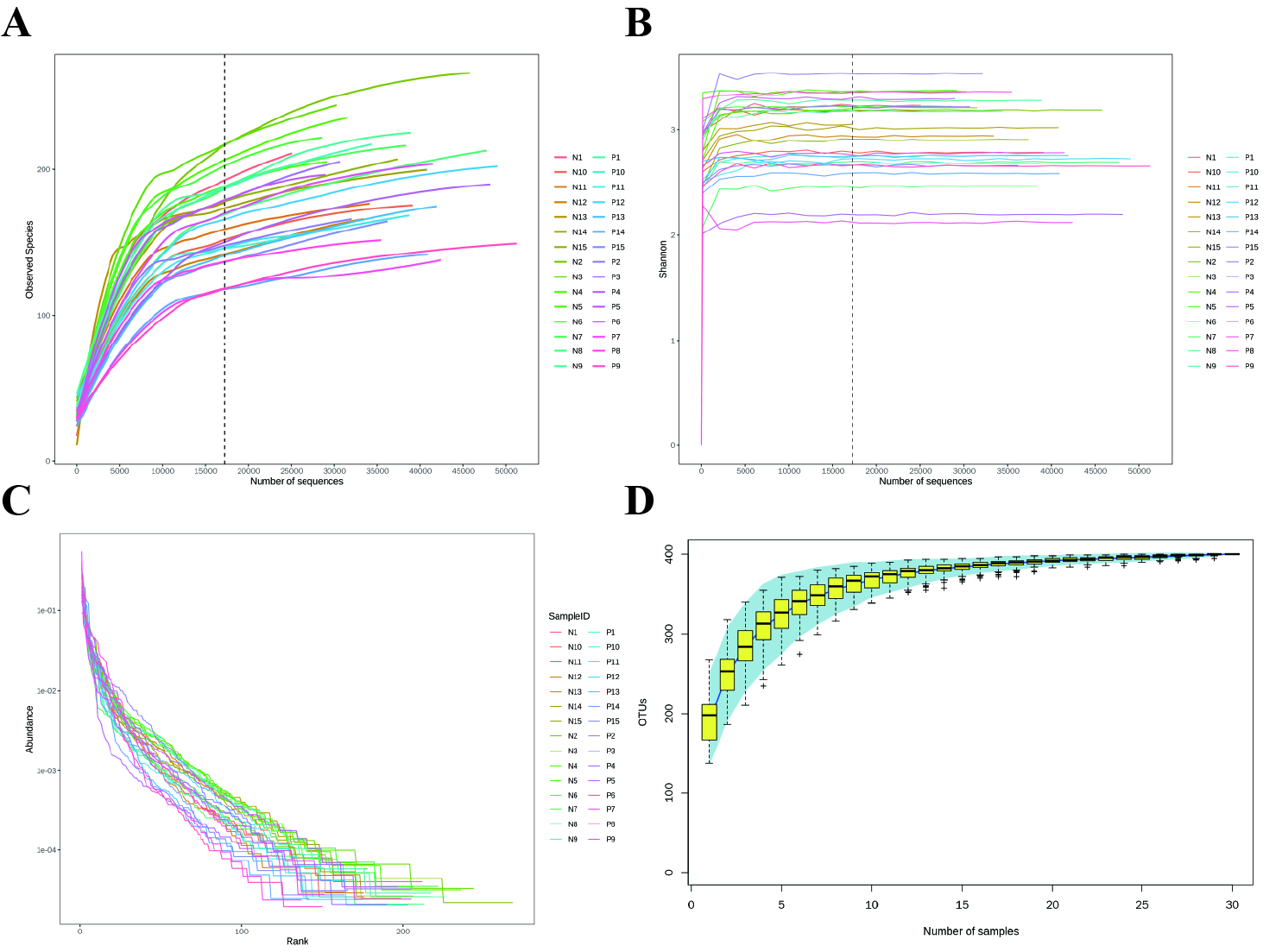

Supplement: Supplementary file 2 — Additional file 2: Figure S2. Test of the adequacy of sample size and data reliability of microbial information; A: Rarefaction curve reflecting the rationality of the data and the richness of species in the sample; B: Shannon curve indicating that the amount of sequencing data is large enough to reflect the vast majority of microbial information in the samples; C: Rank abundance curve reflecting species abundance and uniform distribution of species; D: Species accumulation curve on the adequacy of sample size and estimation of species richness. [file 13099_2022_499_MOESM2_ESM.tif]
